# Modelling a two-stage adult population screen for autosomal dominant familial hypercholesterolaemia: cross-sectional analysis within the UK Biobank

**DOI:** 10.1136/bmjph-2023-000021

**Published:** 2023-10-29

**Authors:** Jasmine Gratton, Steve E Humphries, Amand Floriaan Schmidt, Riyaz S Patel, Reecha Sofat, Chris Finan, Joan K Morris, Aroon D Hingorani, Marta Futema

**Affiliations:** 1 Institute of Cardiovascular Science, University College London, London, UK; 2 Department of Pharmacology and Therapeutics, University of Liverpool, Liverpool, UK; 3 Population Health Research Institute, St George's University of London, London, UK; 4 Cardiology Research Centre, Molecular and Clinical Science Institute, St George's University of London, London, UK

**Keywords:** Public Health, Preventive Medicine, Primary Prevention

## Abstract

**Background:**

Most people with autosomal dominant familial hypercholesterolaemia (FH) remain undetected, which represents a missed opportunity for coronary heart disease prevention.

**Objective:**

To evaluate the performance of two-stage adult population screening for FH.

**Design:**

Using data from UK Biobank, we estimated the screening performance of different low-density lipoprotein cholesterol (LDL-C) cut-offs (stage 1) to select adults for DNA sequencing (stage 2) to identify individuals with FH-causing variants in *LDLR, APOB, PCSK9* and *APOE*. We estimated the number of additional FH cases detected by cascade testing of first-degree relatives of index cases and compared the overall approach with screening in childhood.

**Setting:**

UK Biobank.

**Participants:**

140 439 unrelated participants of European ancestry from UK Biobank with information on circulating LDL-C concentration and exome sequence.

**Main outcome measures:**

For different LDL-C cut-offs, we estimated the detection and false-positive rate, the proportion of individuals who would be referred for DNA sequencing (stage 1 screen positive rate), and the number of FH cases identified by population screening followed by cascade testing.

**Results:**

We identified 488 individuals with an FH-causing variant and 139 951 without (prevalence 1 in 288). An LDL-C cut-off of >4.8 mmol/L had a stage 1 detection rate (sensitivity) of 40% (95% CI 36 to 44%) for a false-positive rate of 10% (95% CI 10 to 11%). Detection rate increased at lower LDL-C cut-offs but at the expense of higher false-positive and screen positive rates, and vice versa. Two-stage screening of 100 000 adults using an LDL-C cut-off of 4.8 mmol/L would generate 10 398 stage 1 screen positives for sequencing, detect 138 FH cases and miss 209. Up to 207 additional cases could be detected through *two-generation* cascade testing of first-degree relatives. By comparison, based on previously published data, childhood screening followed by cascade testing was estimated to detect nearly three times as many affected individuals for around half the sequencing burden.

**Conclusions:**

Two-stage adult population screening for FH could help achieve the 25% FH case detection target set in the National Health Service Long Term Plan, but less efficiently than childhood screening and with a greater sequencing requirement.

**WHAT IS ALREADY KNOWN ON THIS TOPIC**

- Familial hypercholesterolaemia (FH) affects around 1:250–300 individuals in the population and is still highly underdiagnosed. Affected individuals are at increased risk of premature coronary heart disease and benefit from early treatment with low-density lipoprotein cholesterol (LDL-C) lowering therapies. The National Health Service (NHS) Long Term Plan aims to detect 25% of FH cases over the next few years but has not outlined a strategy for doing so. Childhood screening of FH has been evaluated but was turned down by the National Screening Committee.

**WHAT THIS STUDY ADDS**

- Adult screening for FH could help achieve the NHS Long Term Plan goal of detecting more cases in the general population but is less efficient than childhood screening.

**HOW THIS STUDY MIGHT AFFECT RESEARCH, PRACTICE OR POLICY**

- Two-stage adult screening for FH could be implemented as a national screening programme to identify at-risk individuals and reduce the burden of premature coronary heart disease in the general population. Policy-makers will need to consider the cost and efficiency of adult versus childhood screening in deciding which route to follow.

## Introduction

 Autosomal dominant familial hypercholesterolaemia (FH) is caused by a heterozygous DNA variant in either *LDLR*, *APOB, PCSK9* or *APOE* genes, leading to defective clearance of low-density lipoprotein cholesterol (LDL-C).^1^,^5^ Affected individuals have an increased risk of coronary heart disease (CHD) at all ages: standardised incidence ratios for CHD in men and women are 11.1 and 17.3 at ages 25–39, 6.7 and 8.7 at 40–49, 3.3 and 4.5 at 50–59, and 3.3 and 3.1 at 60–69.^6^ Once diagnosed, especially at a young age, people with FH can benefit from drugs to lower LDL-C and reduce the risk of a coronary event.^7^,^9^

Adults with autosomal dominant FH have a higher risk of CHD than people who have a similar LDL-C but without a causative genetic variant.^10^ Moreover, first-degree relatives of people with autosomal dominant FH have a one in two chance of carrying the same causative genetic variant and can be identified by cascade testing in families of index cases.^11^,^13^ Because FH cases are currently identified opportunistically rather than systematically, either when presenting with CHD at a young age, or after an incidental finding of an elevated LDL-C concentration, cascade testing is constrained by the small number of index cases identified.

Consequently, FH is highly underdiagnosed worldwide.^14^ In the UK, only 19 000 (7%) of the estimated 270 000 FH cases are known.^15 16^ The National Health Service (NHS) Long Term plan sets a target of detecting at least 25% of FH cases (~49 000 additional cases) over the next 5 years, but does not specify a screening strategy.^17^ Measurement of circulating LDL-C concentration in adults performs poorly when used alone in distinguishing people with an FH genetic variant from those with a high LDL-C due to diet, lifestyle, or carriage of a high burden of common genetic variants that affect LDL-C concentration.^18 19^ LDL-C concentration in children differentiates people with FH more accurately than in adults and underpins the concept of childhood screening followed by cascade testing.^12^ In the latter approach, children are screened by the age of 2 years by measurement of LDL-C, followed by genetic testing of stored samples with an LDL-C beyond a prespecified cut-off (‘reflex screening’).^20 21^ Affected parents, older siblings and grandparents (three generations) are then identified by cascade testing in families of affected children.

The feasibility and efficiency of childhood screening was reported previously,^20^ and several countries are now running pilot studies.^22^ However, a 2019, review by UK National Screening Committee concluded screening is not recommended in childhood, mainly because of uncertainty regarding the long-term benefit and the age at which screening should occur though these concerns were countered.^23 24^ Statin treatment in children carrying an FH variant should be considered by the age of 8 years according to the European recommendations, or by the age of 10 years as per UK National Institute for Health and Care Excellence (NICE) guidelines.^9 25^

Different genes and DNA variants will cause FH in different families. Thus, sequencing of the four relevant FH-causing genes is needed to identify the causative variant in an index case after which cheaper single-mutation detection methods can be employed for cascade testing of family members.^26^ Although DNA sequencing is more accurate than biochemical screening, and could be used at any age, it is currently too expensive to be considered as the primary screening method for FH.

An approach that minimises sequencing burden while avoiding concern about FH screening in childhood is a two-stage adult screening design. LDL-C, an inexpensive but unspecific test is measured at stage 1, followed by sequencing of FH genes (stage 2) but only in those whose LDL-C concentration exceeds a specified cut-off, mitigating the currently high cost and limited availability of sequencing technologies. However, the performance of two-stage adult screening has not been evaluated or compared with childhood screening.

Participants in UK Biobank, a national, population-based cohort study, have already had LDL-C measurement and whole exome sequencing, which offers an opportunity to model the performance of two-stage adult population screening for FH. The age range of UK Biobank participants at recruitment overlaps with that of individuals who, until the COVID-19 pandemic, were invited to NHS Health Checks in England.^27^ NHS Health Checks evaluate a range of cardiovascular risk factors and blood is routinely drawn for the measurement of circulating lipid concentration. Since genomic sequencing could subsequently be undertaken from a stored blood sample in those with an LDL-C above a prespecified cut-off (adult reflex testing), the NHS Health Check programme, if it continues, could serve as a possible setting for an adult FH screening programme.

Here, we model the performance of two-stage adult population screening to identify index FH cases and compare it with the previously reported performance of two-stage childhood screening for FH.^12 20^

## Methods

### Participants

UK Biobank recruited ~500 000 participants between 40 and 75 years of age, between 2006 and 2010.^28^ Participants completed questionnaires, undertook a variety of physical assessments, and provided biological samples for genotyping, sequencing and other measurements.^29^ The current study is a cross-sectional analysis within UK Biobank. We compared demographic characteristics of UK Biobank participants whose data were analysed in this study with those of individuals enrolled into the NHS Vascular Health Checks in 2017–2018 using data from a previous report.^30^

### LDL-C measurement

In a total of 486 459 UK Biobank participants, serum was obtained from a blood draw in the non-fasting state at the time of recruitment and stored at −80°C and liquid nitrogen for later analysis. LDL-C was measured directly by enzymatic protective selection analysis with a Beckman Coulter AU5800 and the values were recorded in mmol/L.^31^ The choice of the instrument for LDL-C measurement and procedures around this process have been decided by the UK Biobank committee, with the main aim to minimise and mitigate the effects of error (both systematic bias and random error) and to provide high-quality biomarker data.

Where an LDL-C measurement was missing for an included participant (7007 participants in total), we imputed it using single imputation with the R package MICE V.3.10.0.^32^ Where an included participant was already recorded as receiving a statin, we adjusted their LDL-C concentration using the correction coefficient 1.43.^33^

### Identification of carriers of FH-causing genetic variants

A blood sample was drawn for DNA analysis in 454 787 participants of UK Biobank and stored at −80°C.^34^ Exome capture was done using the IDT xGen Exome Research Panel V.1.0, and exome-sequencing was performed on the Illumina NovaSeq 6000 platform.^35^

We identified 140 439 European ancestry participants from data-field 22 006 of the UK Biobank at the time of analysis with whole exome sequence data and valid (directly typed or imputed) measurements of LDL-C and included them in the study. Each participant was assigned to one of three groups: (1) individuals with an established FH-causing variant in *LDLR*, *APOB,* or *PCSK9* genes, or the p.Leu167del variant in *APOE* (online supplemental tables 1 and 2)^36^; (2) individuals with variants of unknown significance (VUS) in *LDLR*, *APOB* or *PCSK9* (online supplemental table 3); and (3) individuals with no FH causing variant or VUS. We classified individuals from the first group as ‘affected’ and those from the other two groups as ‘unaffected’. Further details of the annotation and classification of FH variants is provided in online methods.

### Evaluation of two-stage adult FH screening performance

We counted the number of individuals with an FH-causing variant above and below different LDL-C cut-off values and used this to estimate the stage 1 detection rate (the proportion of eligible participants with an FH-causing variant whose LDL-C value exceeded the cut-off (sensitivity)), the stage 1 false-positive rate (the proportion of eligible participants with no FH-causing variant whose LDL-C value exceeded the cut-off (1-specificity)), the odds of being affected given a positive result (the ratio of true to false positives), and the stage 1 screen positive rate (the proportion of individuals whose LDL-C exceeded the cut-off regardless of FH-causing variant status). We assumed that all individuals with an LDL-C value above the cut-off would undergo targeted sequencing (stage 2). We assumed that targeted sequencing has a 100% detection rate for individuals with FH-causing variants, and that individuals with a VUS identified on sequencing would not be taken forward into the cascade testing phase.

### Comparison of screening performance of two-stage adult and childhood screening

We compared the number of samples requiring sequencing, index FH cases detected (and missed) between adult screening in the present study and childhood screening using information from previous reports.^11 12 20 37^ We assumed that samples with an LDL-C concentration beyond a prespecified cut-off would undergo targeted sequencing of FH-causing genes, with a 100% detection rate and 0% false-positive rate.

### Modelling cascade testing in families of index cases

#### Adult screening

Using previously described methods,^11 20^ we estimated the number of additional FH cases that would be identified by cascade testing in families of each adult index case. We assumed cascade testing of first degree relatives only, and that each index case has one sibling and two offspring on average.^38^ We also assumed that cascade testing was limited to two generations since each index case would be between 40 and 75 years of age and so their parents may not be available for testing.

#### Childhood screening

Using the same assumptions about family structure, but assuming that parents *and* grandparents of affected children would be available (three generation testing), we compared the number of affected family members that would be identified by population screening in children compared with adults. We estimated the number of people that need to be screened in adulthood versus childhood to achieve the NHS Long Term Plan target of identifying 25% of FH cases. We assumed that none of the cases identified by cascade testing was previously identified in the population screening phase. However, we note that Morris *et al* have previously accounted for the fact that a proportion of cases identified through cascade testing would have previously been identified in the population screening phase.^11^

### Statistical analyses

All the analyses were performed in R V.4.0.2. The p values of group differences in table 1 were calculated using the Kruskal-Wallis Rank sum non-parametric test for continuous variables, and the Fisher’s Exact Test for count data.

**Table 1:** Characteristics of the study participants

	No FH-causing variant	FH causing variant	P value of group differences
n	139 291	488	
*LDLR* variant (%)	0 (0.0)	374 (76.6)	<0.001
*APOB* variant (%)	0 (0.0)	101 (20.7)	<0.001
*APOE* variant (%)	0 (0.0)	13 (2.7)	<0.001
Age (median (IQR))	58 (51–63)	58 (51–63)	0.803
Sex (male) (%)	63 382 (45.5)	207 (42.4)	0.187
BMI, kg/m^2^ (median (IQR))	26.7 (24.1–29.8)	27.1(23.9–29.8)	0.689
Townsend Deprivation Index (median (IQR))	2.4 (–3.8 to 0.0)	2.2 (–3.7 to 0.2)	0.346
Smoking status (%)			0.827
Non-smoker	79 618 (57.2)	281 (57.6)	
Former smoker	51 177 (36.7)	173 (35.5)	
Light smoker (<10 cigarettes/day)	2021 (1.5)	7 (1.4)	
Moderate smoker (10–19 cigarettes/day)	3497 (2.5)	13 (2.7)	
Heavy smoker (>20 cigarettes/day)	2978 (2.1)	14 (2.9)	
Statin use (%)	18 139 (13.0)	165 (33.8)	<0.001
Family history of CHD (%)	67 013 (48.1)	306 (62.7)	<0.001
Blood biomarkers			
LDL-C (unadjusted), mmol/L (median (IQR))	3.5 (3.0–4.1)	3.9 (3.2–4.8)	<0.001
LDL-C (adjusted for statin users), mmol/L (median (IQR))	3.7 (3.1–4.2)	4.4 (3.7–5.4)	<0.001
Total cholesterol, mmol/L (median (IQR))	5.7 (4.9–6.4)	6.1 (5.2–7.3)	<0.001
Triglycerides, mmol/L (median (IQR))	1.5 (1.1–2.2)	1.3 (0.9–1.9)	<0.001
HDL-C, mmol/L (median (IQR))	1.4 (1.2–1.7)	1.4 (1.2–1.6)	0.086
Disease prevalence			
CHD prevalence (%)	3890 (2.8)	40 (8.2)	<0.001
CVD prevalence (%)	5686 (4.1)	45 (9.2)	<0.001
Type 2 diabetes prevalence (%)	3593 (2.6)	11 (2.3)	0.757

Missing (%) refers to the proportion of missing data in each field.

BMIbody mass indexCHDcoronary heart diseaseCVDcardiovascular disease (defined as CHD, ischaemic and haemorrhagic stroke, heart failure, and atrial fibrillation)FHfamilial hypercholesterolaemiaHDL-Chigh-density lipoprotein cholesterolLDL-Clow-density lipoprotein cholesterol

### Patient and public involvement

Regular public involvement events for UK Biobank participants are organised by UK Biobank. We did not conduct any further activities involving patients or public for this particular study.

## Results

### Demographic and other characteristics of UK Biobank participants

We identified 140 439 white British participants from UK Biobank with a valid (directly measured or imputed) LDL-C value and whole exome sequencing data available at the time of analysis. The baseline characteristics of the analysed UK Biobank participants (median age 58 years, 45% male) are shown in online supplemental table 4. The analysed UK Biobank participants were slightly older than individuals evaluated in the NHS Health Check in 2017–2018 but had a similar gender distribution (online supplemental table 4).^30^ About 16% of those undergoing NHS Health Checks self-reported as non-white, whereas the dataset we analysed from UK Biobank was limited to those of who self-reported as being white (online supplemental table 4).^30^

### UK Biobank participants with an FH-causing variant or VUS

**Figure 1:**
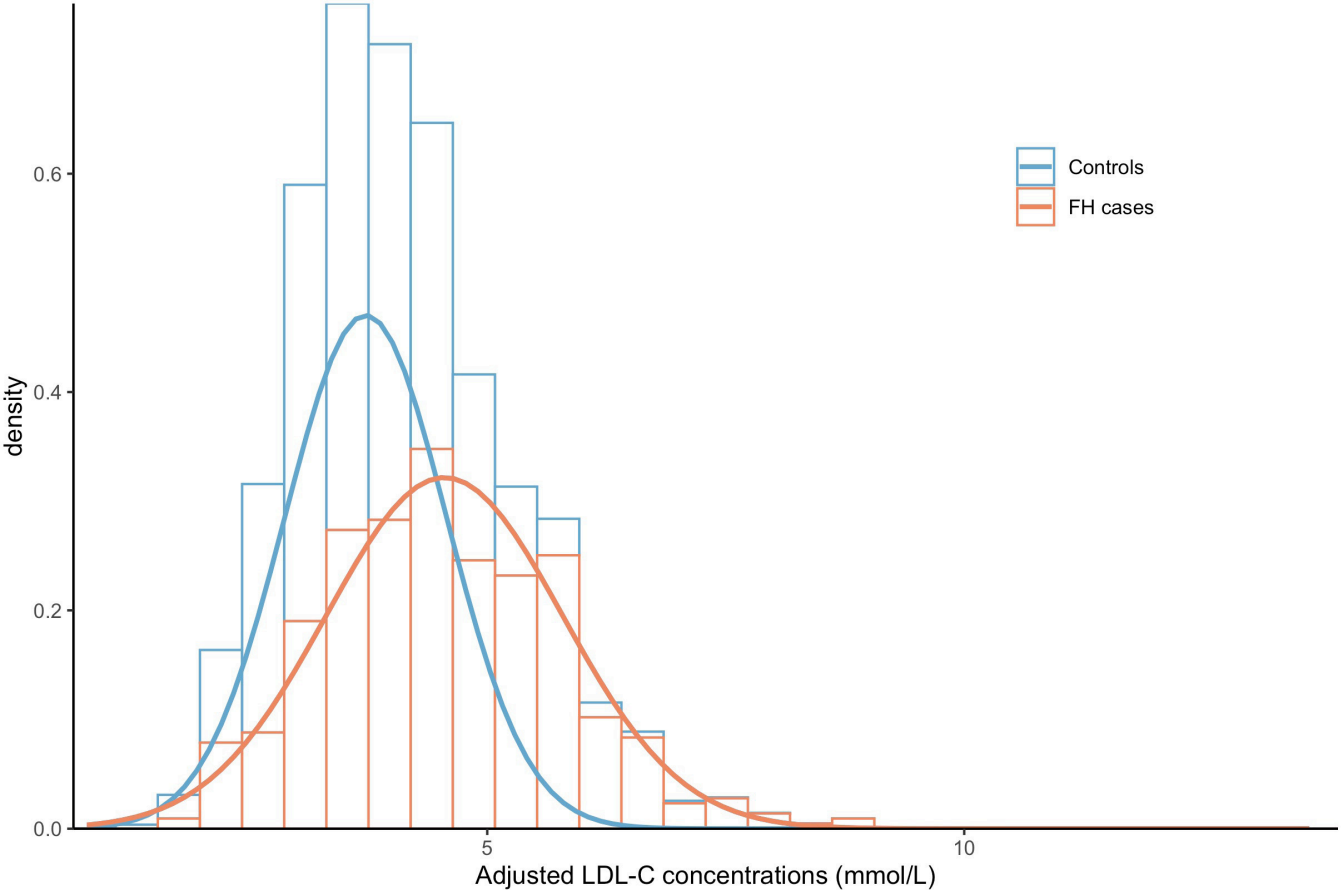
Distributions of the adjusted low-density lipoprotein cholesterol (LDL-C) concentrations in monogenic familial hypercholesterolaemia (FH) carriers and non-carriers of the study cohort. Unaffected individuals are shown in blue and affected individuals in red. The histograms represent the distribution of the data, and the smoothed distributions are constructed based on the mean and SD of the affected and unaffected participants. The means of the distributions are represented by the dotted vertical lines.

Of the 140 439 participants studied, 488 had an FH-causing variant interpreted as ‘pathogenic’ or ‘likely pathogenic’ according to American College of Medical Genetics and Genomics guidelines,^39^ giving a prevalence of 1 in 288, which is similar to previous reports.^1^ A further 660 individuals were found to carry a VUS. Of the FH-causing variants, 374 were located in *LDLR* (1 in 376), 101 in *APOB* (1 in 1390), and 13 were the p.Leu167del in *APOE* (1 in 10 803). None of those analysed carried an FH-causing variant in *PCSK9*. A full list of FH-causing variants and VUS is provided in online supplemental tables 2 and 3. LDL-C concentration was higher in those with an FH-causing variant (median 4.43 mmol/L, IQR (3.67–5.43)) than those without (median 3.67 mmol/L, IQR (3.14–4.24)) (figure 1 and table 1). Median LDL-C concentration of *APOE* p.Leu167del variant carriers was 3.68 mmol/L (IQR (3.55–4.98)) which was lower than the LDL-C concentration in carriers of FH-causing variants in *APOB* and *LDLR* (online supplemental table 5). FH-causing variant carriers had significantly lower triglyceride concentration than non-carriers (median 1.3 mmol/L, IQR (0.9–1.9) vs 1.5 mmol/L, IQR (1.1–2.2), p<0.001), which is in line with recent findings that low triglyceride concentration was found to be one of the major predictors of monogenic FH.^40^

There was no significant difference between those with and without FH-causing variants in age, sex, body mass index, Townsend deprivation index, smoking status, high-density lipoprotein cholesterol or lipoprotein(a) concentration (table 1). Of those with FH-causing variants, 34% were on statins, compared with 13% of those without FH-causing variants (p<0.001) (table 1). There was a higher proportion of people with a family history of CHD in those with FH-causing variants than those without (63% vs 48%; p<0.001), as well as more than double the prevalence of both CHD (8% vs 3%; p<0.001) and cardiovascular disease (CVD) (a composite of CHD, ischaemic and haemorrhagic stroke, heart failure and atrial fibrillation) (9% vs 4%; p<0.001) (table 1).

### Performance of two-stage adult screen for autosomal dominant FH

For a range of LDL-C cut-offs between 3 and 8.5 mmol/L, we estimated the detection and false-positive rate of stage 1 screening, the proportion of samples that would be referred for sequencing (stage 1 screen positive rate), and the number and proportion of FH cases identified by the two-stage screen (online supplemental table 6 and figure 1).

**Figure 2:**
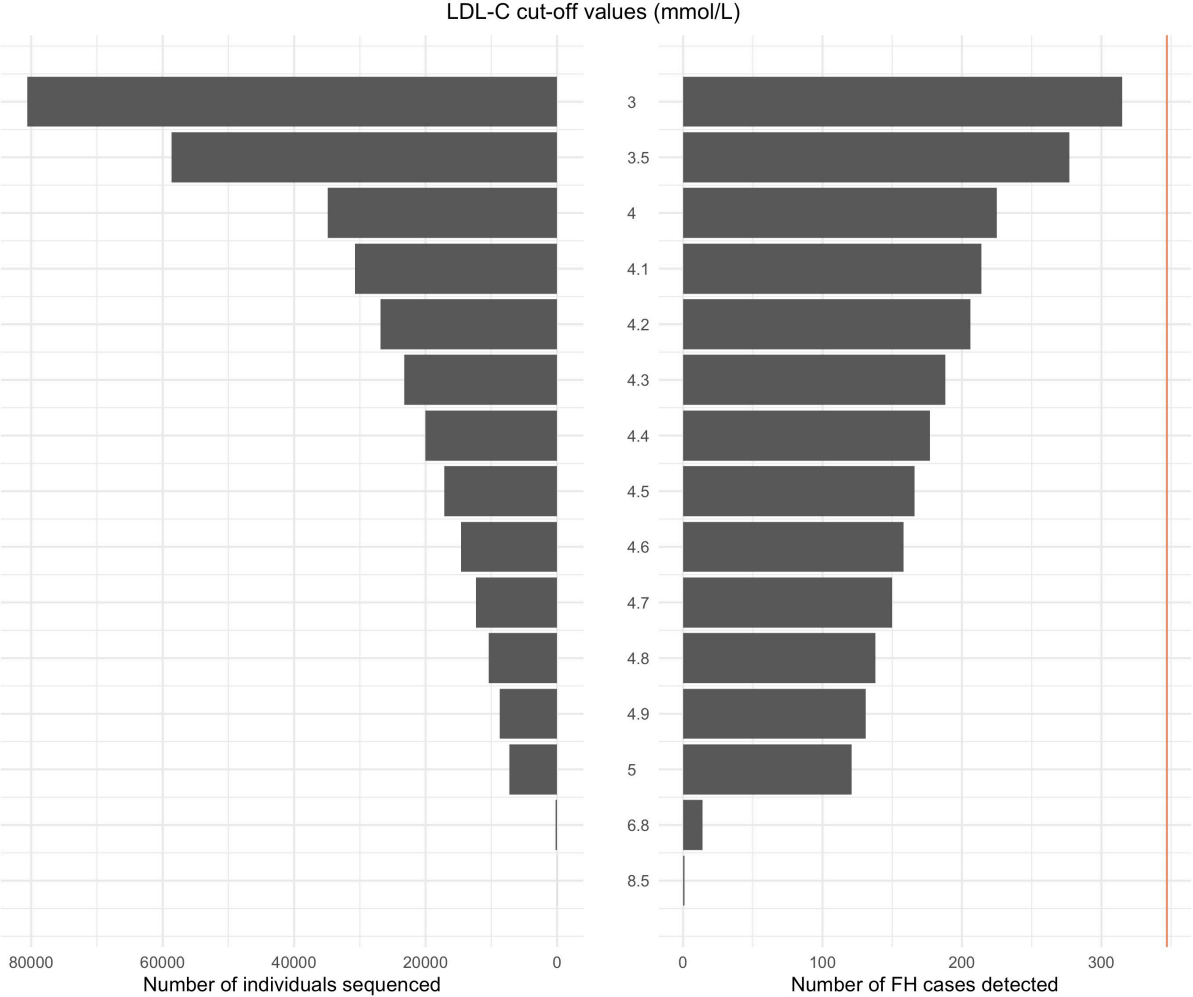
The number of samples sequenced, and the number of familial hypercholesterolaemia (FH) cases detected using various low-density lipoprotein cholesterol (LDL-C) cut-off values in the adult reflex screening population strategy for FH for a hypothetical population of 100 000 individuals. The orange vertical line represents the total number of 347 FH cases in the hypothetical sample population of 100 000 individuals (for an FH prevalence of 1:288).

Selecting a particular LDL-C cut-off for the stage 1 screening involves a tradeoff between detection and false-positive rates. The lower the LDL-C cut-off, the higher the detection rate but also the false-positive rate and therefore the number of stage 1 screen positive samples that would be submitted for sequencing (table 2 and figure 2). For example, using an LDL-C cut-off of 5.0 mmol/L gave a detection rate of 35% (95% CI 31% to 39%) for a 7% false-positive rate (95% CI 7% to 7.3%) at stage 1, with a screen positive rate of 7%. An LDL-C cut-off of 4.0 mmol/L gave a stage 1 detection rate of 65% (95% CI 60% to 69%) but at the expense of a 35% (95% CI 35% to 35%) stage 1 false-positive rate and screen positive rate.

**Table 2:** Performance of a two-stage adult population screen for monogenic FH using different stage 1 LDL-C cut-offs

LDL-C cut-off(mmol/L)	Detection rate (%)	False-positive rate (%)	Stage 1 (LDL-C)					Stage 2 (Sequencing)	
			OAPR	Cases detected	Cases missed	False positives	Number sequenced	Cases confirmed	VUS
3	91 (88–93)	81 (80–81)	1:255	315	32	80 258	80 573	315	417
3.5	80 (76–83)	59 (58–59)	1:211	277	70	58 346	58 623	277	331
4	65 (60–69)	35 (35–35)	1:154	225	122	34 678	34 903	225	218
4.1	62 (57–66)	31 (30–31)	1:143	214	133	30 556	30 770	214	196
4.2	59 (55–64)	27 (27–27)	1:129	206	141	26 628	26 834	206	177
4.3	54 (50–59)	23 (23–23)	1:123	188	159	23 075	23 263	188	159
4.4	51 (46–55)	20 (20–20)	1:112	177	170	19 835	20 012	177	139
4.5	48 (43–52)	17 (17–17)	1:102	166	181	16 997	17 163	166	124
4.6	46 (41–50)	15 (14–15)	1:92	158	189	14 470	14 628	158	108
4.7	43 (39–48)	12 (12–12)	1:81	150	197	12 195	12 345	150	92
4.8	40 (36–44)	10 (10–11)	1:74	138	209	10 260	10 398	138	79
4.9	38 (34–42)	9 (9-9)	1:65	131	216	8572	8703	131	70
5	35 (31–39)	7 (7-7)	1:59	121	226	7116	7237	121	59
6.8	4 (3-6)	0.2 (0.2–0.2)	1:15	14	333	211	225	14	1
8.5	0.4 (0.1–2)	0 (0–0)	1:15	1	346	15	16	1	0

Reported counts are based on a screened population of 100 000 adults with 347 monogenic FH cases and 470 individuals with a VUS.

FHfamilial hypercholesterolaemiaLDL-Clow-density lipoprotein cholesterolOAPRodds of being affected given a positive test resultVUSvariant of uncertain significance

**Figure 3:**
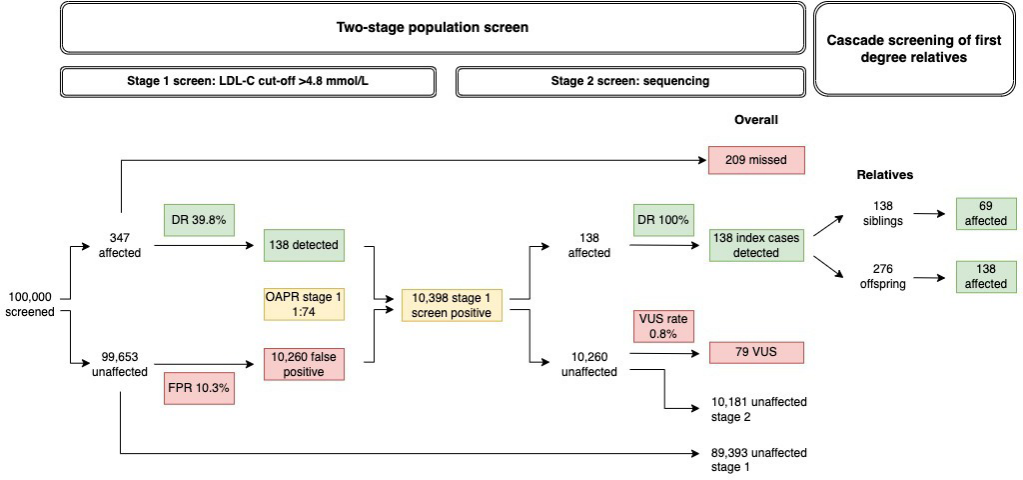
Illustration of the two-stage screen and subsequent cascade screening of first-degree relatives of index FH cases scaled to 100 000 individuals using an LDL-C cut-off value of 4.8 mmol/L in the first stage screen. DR, detection rate (sensitivity); FPR, false-positive rate (1-specificity); OAPR, odds of being affected given a positive result; VUS, variant of uncertain significance.

For illustration, figure 3 shows the performance of the two-stage screen scaled to a cohort of 100 000 people, using a stage 1 LDL-C cut-off of 4.8 mmol/L selected to provide a reasonable trade-off between detection and false-positive rates. The 10 398 stage 1 screen positive individuals include 138 (40%) of the expected 347 FH cases, all of whom would be identified by sequencing at stage 2. Individuals who are screen positive from stage 1 would also include 10 181 individuals with no FH-causing variant or VUS, as well as 79 individuals with a VUS; the two groups together giving a stage 1 false-positive rate of 10% (95% CI 10% to 11%). All these individuals would be correctly classified by DNA sequencing at stage 2, giving a stage 2 false-positive rate of 0%, with a VUS rate of 0.8%. Table 2 documents the corresponding values for the detection, false-positive and screen-positive rates for different LDL-C cut-offs.

### Comparison of two-stage adult and childhood screening

Wald *et al* previously evaluated the performance of Child–Parent Familial Hypercholesterolemia Screening in Primary Care.^20^ They screened 10 095 children. A total of 37 children were eventually found to have an FH mutation: 30 of these were identified by mutation testing using a genotyping panel, and a further 7 children, those with a total cholesterol (TC)>1.53 multiples of the median value (MoM), were identified using sequencing. This gave a prevalence of mutation positive FH of 1 in 273, slightly higher than we identified in the UK Biobank cohort in the current analysis where the prevalence was 1 in 288. Using a test cut-off for TC of 1.35 MoM (the 95th centile for the TC distribution), Wald *et al* found that 28 of the 37 children with an FH mutation had a TC above the cut-off, giving a detection rate of 28/37=76%. A total of 477 of 10 058 children without an FH mutation had a TC greater than 1.35 MoM, giving a false-positive rate of 4.7%. To enable comparison with adult screening, we applied the same test cut-off for TC concentration and the same calculated detection and false-positive rate, but applied it to a hypothetical cohort of 100 000 children in which the prevalence of FH is 1 in 288 (rather than 1 in 273). This equates to 347 affected children of whom 263 would be identified (detection rate=76%) and 84 would be missed.

Two-stage adult screening of 100 000 individuals based on an LDL-C cut-off of >4.8 mmol/L therefore identifies just under half as many index FH cases (138 vs 263) for twice the burden of sequencing (10 398 vs 5000 samples) when compared with childhood screening of 100 000 participants at age 2 years using a TC cut-off of ≥1.35 MoM (table 3).^12 20^ Childhood screening identifies one index case per 380 individuals screened and 19 sequenced, whereas the corresponding values for two-stage adult screening are one index case per 725 screened and 75 sequenced (table 3).

**Table 3:** Comparison of childhood and the two-stage adult screening for FH followed by cascade testing

Population screen	Childhood	Adult two-stage
Target population	Children	Adults
Number screened	100 000	100 000
Estimated FH population prevalence	1 in 288	1 in 288
Estimated FH cases	347	347
Test	Total cholesterol	LDL-C
Test cut-off	≥1.35 MoM (95th centile)	>4.8 mmol/L
Index FH cases missed	84	209
Index FH cases detected	263	138
False positives	4737	10 260
Number eligible for sequencing	5000	10 398
Index FH cases confirmed on sequencing	263	138
Cascade testing (best case scenario)*		
Generations screened	3	2
Affected siblings of index case	132	69
Affected parents of index case	263	Not applicable
Affected grandparents of index case	263	Not applicable
Affected offspring of index case	Not applicable	138
Combined		
Number of FH cases detected	921	345
Screening efficiency		
Number needed to screen to identify one FH case (population screen)	380	725
Number needed to sequence to identify one FH case (population screen)	19	75
Number needed to screen to identify one FH case (combined)	109	290
Number needed to sequence to identify one FH case (combined)	5	30

The FH prevalence of 1:288 of the UK Biobank was applied to the modelled childhood screen and counts were adjusted based on the detection and false-positive rate for childhood screening reported in.^12 20^

* Estimates are based on figures from the Office of National Statistics where the average UK family comprises of two children.^38^ The best case scenario for cascade testing refers to all first degree relatives with FH identified through cascade testing (see reference 12),

FHfamilial hypercholesterolaemiaLDL-Clow-density lipoprotein cholesterolMoMmultiple of the median

### Cascade testing following the two-stage adult and childhood screening

Childhood screening can seed cascade testing of three generations whereas adult two-stage screening at the average age of participants in UK Biobank allows cascade testing of only two generations (assuming parents of an index case are unavailable for screening by virtue of their age).

Cascade testing in the families of the 138 index adult cases would detect 69 affected siblings and 138 affected children on average. Cascade testing in the families of the 263 affected children would identify 263 affected parents of the index child, 263 affected grandparents of index child and 132 affected siblings of index child, on average. In total (including index cases), 921 affected individuals would be identified from screening 100 000 children followed by 3 generation cascade testing in families of index cases. This means that cascade testing following childhood screening has the potential to identify almost three times as many familial cases as adult screening (up to 921 vs 345 cases for 100 000 individuals screened) (table 3). Overall (combining screening and cascade testing of index cases), childhood screening could identify one FH case per 109 individuals screened and 5 sequenced, whereas the corresponding values in adult screening are one FH case per 290 screened and 30 sequenced.

### Achieving the target set in the NHS long-term plan

#### Best case scenario

Under a best-case scenario, and using the simplifying assumption that none of the individuals identified through cascade testing was previously identified in population screening, ~14 million adults would need to be screened and ~1.5 million sequenced to reach the NHS Long Term Plan goal of identifying all 25% of UK FH cases (49 000 additional cases),^17^ whereas only ~5.3 million children would need to be screened and ~5 00 000 sequenced to achieve the same goal. On the assumption that NHS Health Checks screen around 1 million adults annually,^30^ it would take around 14 years to reach this goal. Assuming 2020’s 681 560 live births in England and Wales and a 95% childhood vaccination uptake at 2 years when an LDL-C measurement could be made,^41 42^ around 650 000 children would be eligible for screening each year in the childhood screening strategy, which would achieve the 25% FH case detection rate in 8 years.

#### Realistic scenario

However, the best case scenario is unlikely to be achieved for several reasons: (1) uptake of cascade testing is about 84% and in practice identifies only about one additional FH case per family on average (HEART UK 2022 conference communication)^43 44^; (2) cascade testing reidentifies affected individuals from the population screen; and (3) the best case scenario assumes family members identified through cascade testing are never counted twice. Using a model that accounts for reidentification and double counting, Wald and Bestwick estimated that it would take 8 years for childhood screening followed by cascade testing to identify 25% of FH cases.^45^ Given adult screening is about one-third as efficient as childhood screening but that 1.5 times as many adults as children might be screened each year (1 000 000/650 000; based on health check and vaccination data), achieving the NHS Target through adult population screening might take twice (3×2/3=2) as long as through childhood screening.

## Discussion

We have modelled the performance of a two-stage screen for FH in individuals between the age of 40 and 75 years (median age 58 years) using data available from UK Biobank. The two-stage screen combines an inexpensive test with a high false-positive rate at stage 1 (LDL-C concentration), with an expensive test with low false-positive rate at stage 2 (DNA sequencing). An LDL-C cut-off of 4.8 mmol/L at stage 1 was estimated to detect 40% (95% CI 36% to 44%) of those affected for a false-positive rate of 10% (95% CI 10% to 11%). This cut-off would result in 10 398 samples being sequenced for every 100 000 people screened. Lowering the LDL-C cut-off would increase the number of people with FH who are detected but increase the sequencing burden and vice versa.

If the adult two-stage population screening approach were used to seed a cascade testing programme among first-degree relatives of index cases, this would lead to the detection of a three further affected family members for every two index cases, under the best case scenario.^12^ This means that 1 FH case would be detected for every 290 participants screened instead of 1 case detected for every 725 individuals for the population screen alone.

The approach to screening modelled here is less efficient than childhood screening followed by cascade testing proposed and evaluated previously.^20 21 45^ Two-stage adult screening with two-generation cascade testing identifies about a third as many FH cases as childhood screening with three-generation cascade testing, for twice the sequencing burden. Detecting 25% of all FH cases (~49 000 additional cases; the target set in the NHS Long Term Plan) requires screening around 14 million adults and sequencing of 1.5 million of them, or 5.3 million children and sequencing 500 000 of them, assuming no reidentification and double counting. Despite this efficiency advantage, childhood screening was not endorsed by the National Screening Committee when last reviewed on the grounds that it does not immediately benefit the children who are screened at around 1 year of age.^25^ The developers of the approach have countered this and other concerns that were raised,^23 24^ but at present, childhood screening is not in general use in the UK, though some pilot studies are underway.

The NHS Long-Term Plan has a stated aim of increasing the proportion of detected FH cases from 7% to 25% in the next 5 years, but does not elaborate on how this is to be achieved.^17^ Cascade testing has been endorsed by the NICE (CG 71),^13^ but the efficiency of cascade testing is dependent on the flow of index cases. The approach we have modelled involves a two-stage population screen in adults in which the high false-positive rate of a stage 1 LDL-C measurement is mitigated by the low false-positive rate of a stage 2 DNA sequencing test. Although less efficient than childhood screening, it avoids concerns about screening for FH in childhood raised by the National Screening Committee. Based on estimates here for adult screening and on those made by others for childhood screening, achieving the NHS Long Term plan target of 25% of FH cases detected in the next 5 years seems unrealistic, with childhood screening offering the chance of achieving this target about twice as quickly as adult screening.

Nevertheless, if two-stage adult screening followed by cascade testing for FH were to be the preferred approach several practical issues require consideration including: (1) the potential setting of such a screening programme; (2) the capacity of the NHS Genomic Medicine Service to undertake sequencing on the necessary, scale; and (3) the capacity to undertake cascade testing of first-degree relatives of index cases identified through population screening.

### Potential setting of a two-stage adult screening programme

An NHS Health Check was operating in England since 2009 and was offered to men and women aged 40–74 without previously diagnosed hypertension, diabetes mellitus, FH, CHD, heart failure, atrial fibrillation, stroke or transient ischaemic attack, peripheral arterial disease, or chronic kidney disease. Those already on statins or known to have a 10-year CVD risk of ≥20% were excluded. Of those invited, about 50% attended; about 1 million people per annum, and participants in the NHS Health Check better represent the ethnic mix of the English population.^30^ Thus, if reintroduced, the NHS Health Check could, in principle, provide the setting for a two-stage population screen for FH modelled here.

### DNA sequencing capacity in the NHS

The National Genomic testing service was established to enable the NHS to harness the power of genomic technology and science to improve the health of the population and deliver on the commitments of the NHS Long Term Plan.^46^ One of its stated aims is the ‘early detection and treatment of high-risk conditions including expanding genomic testing for familial hypercholesterolaemia’. If 1 million adults per annum underwent a stage 1 screen via the NHS Health Check, the National Genomic Medicine Service would need to develop capacity to offer targeted sequencing of FH-causing genes in around 100 000 people per annum.

### Cascade testing capacity

NICE Guideline CG71 has already drawn up recommendations for cascade testing in families where an FH-causing variant has been detected in an index case.^13^ Under the current guidance, NICE suggests case finding should be based on identification of individuals whose TC concentration exceeds 7.5 mmol/L below and over 9.0 mmol/L above 30 years of age, which is very high and therefore likely to miss many FH-variant carriers. However, it makes no recommendation on the systematic measurement of cholesterol concentration and implies that potential cases are identified through surveys of existing health records, which is not a comprehensive approach. If one million individuals attend their health check per annum, we estimated 103 980 people would be referred for sequencing after a stage 1 screen for FH using LDL-C; this would likely result in around 1327 index cases being detected which would greatly increase the burden on cascade testing services and likely require investment in workforce and infrastructure to meet this need.

### Limitations

Some limitations of our modelling are noteworthy. We used UK Biobank as a convenience sample because participants (aged between 40 and 75 at the time of recruitment) have had LDL-C measurements and exome sequencing data available for analysis. However, the median age of participants (58 years) is older than might be considered optimal for FH screening adults. Screening at a younger age (eg, 40–50 years) would have the advantage of a potentially higher stage 1 detection rate, because LDL-C concentration better separates FH cases from those with an elevated LDL-C for other reasons at younger than older ages,^19^ and because screening parents of index cases as well as siblings and children may then become possible (three-generation rather than two-generation screening).

Our analysis was also retrospective not prospective. The participants we studied are not representative of the ethnic mix of the UK population, and additional validation of screening performance will be needed, particularly among a sample who better represent the population diversity of England and the devolved nations. The ‘Our Future Health’ programme offers a potential setting for such a validation. Our Future Health has the aim of ‘testing more effective approaches to prevention, earlier detection, and treatment of diseases’.^47^ Our Future Health is aiming to recruit 5 million participants with the aim of testing a range of genomic and other technologies for disease prediction with around 100 000 individuals already expressing interest in participating.^48 49^ Importantly, a strategic aim of Our Future Health is to include a more representative and diverse group of participants than UK Biobank. Thus, a prospective validation of two-stage screening for FH could be embedded within the Our Future Health protocol, with many of the necessary elements already planned and funded and could provide an early key finding from the study.

Classification of some variants, especially VUS, may change in the future, as more evidence about a variant’s effect is gathered and curated by expert panels such as the FH Clinical Genome Resource (ClinGen) consortium.^50^ Should a VUS subsequently be designated as pathogenic, individuals with such a variant would be reclassified as affected. This would increase the detection and reduce the false-positive rate of the two-stage screen and increase the number of cases identified by cascade testing.

In summary, we have used data from UK Biobank to model the performance of two-stage population screening to identify index FH cases and to estimate the performance of cascade screening in affected families. We compared the performance with childhood screening and although we found it to be about half as efficient as childhood screening followed by cascade testing and take twice as long to achieve the target of detecting 25% of FH cases as stated in the NHS Long Term Plan. Nevertheless, if adopted, the approach could be evaluated prospectively through the Our Future Health programme, and if feasible and cost-effective, then the foundations for a national programme may already be in place through the NHS Health Check, the NHS Genomic Medicine Service, and the NICE endorsed frameworks for cascade testing. However, substantial investment in workforce and infrastructure is likely to be needed, which would need to be weighed against the cost and efficiencies of childhood screening.

## supplementary material

10.1136/bmjph-2023-000021 online supplemental file 1

## Data Availability

Data may be obtained from a third party and are not publicly available.
