# Supplementary material for: Modelling a two-stage adult population screen for autosomal dominant familial hypercholesterolaemia: cross-sectional analysis within the UK Biobank
Source: BMJ Public Health. 2023 Oct 29;1(1):e000021. doi: 10.1136/bmjph-2023-000021 (PMC11812690; doi:10.1136/bmjph-2023-000021)

**SUPPLEMENTAL MATERIAL**

## ONLINE METHODS

We annotated FH causing variants as follows. All variants called in *LDLR*, *PCSK9*, *APOE*, and *APOB* genes were extracted from the exome data using the GRCh38 coordinates listed in Supplemental Table 1. Multiallelic sites for single nucleotide polymorphisms (SNPs) and insertions and deletions (indels) were normalised (i.e. converted to biallelic records) with BCFtools version 1.11,[1] and all variants were annotated using Ensembl Variant Effect Predictor (VEP) release 103.1.[2] Individuals with a heterozygous p.Leu167del in-frame deletion in the *APOE* gene were considered to be FH-variant positive.[3] FH-causing variants in the *PCSK9* and *APOB* genes were selected based on a list of curated variants that have been validated in functional assay.[4] Variants in the *LDLR* gene were filtered by minor allele frequency (MAF) of <0.0006, which is the frequency of the single most common FH mutation, the p.Arg3527Gln in *APOB*, as reported in the gnomAD database.[5] Sequencing quality filters were applied using BCFtools: variants with a read depth of less than 10, a genotype quality of less than 20 were removed. We used the canonical transcript ENST00000558518 for *LDLR*, and the SAMtools plugin split-vep was used to further filter the variants.[1] *LDLR* variants were retained if they had a predicted consequence of missense or more severe, and were excluded if they had a SIFT annotation matching "tolerated" or a PolyPhen entry matching "benign".[6,7] Heterozygous and homozygous variants were manually curated by two independent expert reviewers (M.F. and S.E.H.), who followed the Association for Clinical Genomics Science (ACGS) guidelines for variants classification and the evidence curated in the gene specific database.[8,9] A full list of the FH-causing variants identified can be found in Supplemental Table 2. A further 660 individuals were identified variants of uncertain significance (VUS) in *LDLR*, *PCSK9* and *APOB* genes (listed in Supplemental Table 3).

## REFERENCES


1 Danecek P, Bonfield JK, Liddle J, *et al.* Twelve years of SAMtools and BCFtools. *Gigascience* 2021;**10**. doi:10.1093/GIGASCIENCE/GIAB008

2 McLaren W, Gil L, Hunt SE, *et al.* The Ensembl Variant Effect Predictor. *Genome Biol 2016 171* 2016;**17**:1–14. doi:10.1186/S13059-016-0974-4

3 Awan Z, Choi HY, Stitziel N, *et al.* APOE p.Leu167del mutation in familial hypercholesterolemia. *Atherosclerosis* 2013;**231**:218–22. doi:10.1016/J.ATHEROSCLEROSIS.2013.09.007

4 Lázaro C, Lerner-Ellis J, Spurdle A. *Clinical DNA variant interpretation theory and practice*. Academic Press 2021.

5 Karczewski KJ, Francioli LC, Tiao G, *et al.* The mutational constraint spectrum quantified from variation in 141,456 humans. *Nat 2020 5817809* 2020;**581**:434–43. doi:10.1038/s41586-020-2308-7

6 Adzhubei IA, Schmidt S, Peshkin L, *et al.* A method and server for predicting damaging missense mutations. *Nat Methods 2010 74* 2010;**7**:248–9. doi:10.1038/nmeth0410-248

7 Kumar P, Henikoff S, Ng PC. Predicting the effects of coding non-synonymous variants on protein function using the SIFT algorithm. Published Online First: 2009. doi:10.1038/nprot.2009.86

8 Ellard S, Baple EL, Callaway A, *et al.* ACGS Best Practice Guidelines for Variant Classification in Rare Disease 2020 Recommendations ratified by ACGS Quality Subcommittee on 4 th. Published Online First: 2020. doi:10.1101/531210

9 Fokkema IFAC, Taschner PEM, Schaafsma GCP, *et al.* LOVD v.2.0: the next generation in gene variant databases. *Hum Mutat* 2011;**32**:557–63. doi:10.1002/HUMU.21438

10 NHS Health Check programme, Patients Recorded as Attending and Not Attending, 2012-13 to 2017-18 - NHS Digital. https://digital.nhs.uk/data-and-information/publications/statistical/nhs-health-check-programme/2012-13-to-2017-18 (accessed 3 Mar 2022).

**Supplemental Table 1. Genetic coordinates of the *LDLR*, *APOE*, *APOB* and *PCSK9* genes.** The genetic coordinates used to extract the FH-causing genes from the whole exome sequencing data are mapped to GRCh38.

| Gene name | Chromosome number | Start coordinate | End coordinate |
|---|---|---|---|
| *LDLR* | 19 | 11,089,262 | 11,133,820 |
| *APOB* | 2 | 21,001,429 | 21,044,073 |
| *APOE* | 19 | 44,905,791 | 44,909,393 |
| *PCSK9* | 1 | 55,039,347 | 55,064,852 |

**Supplemental Table 2. Autosomal dominant FH-causing mutation identified in our study cohort.** Genetic coordinates are mapped to GRCh38.

| Gene | Chromosome | Position | Reference allele | Alternate allele | Nucleotide change | Protein | Number of carriers | UKB frequency (1/n) |
|---|---|---|---|---|---|---|---|---|
| *APOB* | 2 | 21006289 | G | A | c.10579C>T | p.Arg3527Trp | 2 | 70,220 |
| | | 21006288 | C | T | c.10580G>A | p.Arg3527Gln | 99 | 1,419 |
| *APOE* | 19 | 44908791 | GCTC | G | c.499_501del | p.Leu167del | 13 | 10,803 |
| *LDLR* | 19 | 11100236 | C | G | c.81C>G | p.Cys27Trp | 1 | 140,439 |
| | | 11100291 | T | G | c.136T>G | p.Cys46Gly | 1 | 140,439 |
| | | 11100294 | G | A | c.139G>A | p.Asp47Asn | 5 | 28,088 |
| | | 11102705 | C | T | c.232C>T | p.Arg78Cys | 13 | 10,803 |
| | | 11102714 | C | T | c.241C>T | p.Arg81Cys | 2 | 70,220 |
| | | 11102732 | T | G | c.259T>G | p.Trp87Gly | 6 | 23,407 |
| | | 11102741 | G | A | c.268G>A | p.Asp90Asn | 5 | 28,088 |
| | | 11102765 | G | A | c.292G>A | p.Gly98Ser | 10 | 14,044 |
| | | 11102774 | G | A | c.301G>A | p.Glu101Lys | 12 | 11,703 |
| | | 11102787 | G | A | c.313+1G>A | . | 5 | 28,088 |
| | | 11102787 | G | C | c.313+1G>C | . | 1 | 140,439 |
| | | 11102787 | G | GT | c.313+2dup | . | 2 | 70,220 |
| | | 11105249 | C | T | c.343C>T | p.Arg115Cys | 2 | 70,220 |

| | | | | | | | | |
|---|---|---|---|---|---|---|---|---|
| | | 11105268 | G | T | c.362G>T | p.Cys121Phe | 2 | 70,220 |
| | | 11105324 | G | A | c.418G>A | p.Glu140Lys | 1 | 140,439 |
| | | 11105339 | GTGCTCACCTGTGGTC CCGCCAGC | G | c.435_457del | p.Leu146ProfsTer 26 | 1 | 140,439 |
| | | 11105407 | C | A | c.501C>A | p.Cys167Ter | 2 | 70,220 |
| | | 11105408 | G | A | c.502G>A | p.Asp168Asn | 14 | 10,031 |
| | | 11105415 | AC | A | c.513del | p.Asp172ThrfsTer 34 | 1 | 140,439 |
| | | 11105448 | C | G | c.542C>G | p.Pro181Arg | 2 | 70,220 |
| | | 11105549 | C | T | c.643C>T | p.Arg215Cys | 4 | 35,110 |
| | | 11105567 | G | A | c.661G>A | p.Asp221Asn | 2 | 70,220 |
| | | 11105568 | A | G | c.662A>G | p.Asp221Gly | 5 | 28,088 |
| | | 11105585 | GAC | G | c.680_681del | p.Asp227GlyfsTer 12 | 4 | 35,110 |
| | | 11105585 | GAC | GAG | c.681delinsG | p.Asp227Glu | 2 | 70,220 |
| | | 11105588 | G | T | c.682G>T | p.Glu228Ter | 2 | 70,220 |
| | | 11105589 | AG | A | c.685del | p.Glu229LysfsTer 36 | 1 | 140,439 |
| | | 11106579 | C | T | c.709C>T | p.Arg237Cys | 1 | 140,439 |
| | | 11106588 | G | A | c.718G>A | p.Glu240Lys | 20 | 7,022 |
| | | 11106592 | T | C | c.722T>C | p.Phe241Ser | 1 | 140,439 |
| | | 11106631 | A | C | c.761A>C | p.Gln254Pro | 1 | 140,439 |

| | | | | | | | | |
|---|---|---|---|---|---|---|---|---|
| | | 11107432 | C | A | c.858C>A | p.Ser286Arg | 1 | 140,439 |
| | | 11107433 | G | A | c.859G>A | p.Gly287Ser | 4 | 35,110 |
| | | 11107436 | G | A | c.862G>A | p.Glu288Lys | 1 | 140,439 |
| | | 11107461 | G | A | c.887G>A | p.Cys296Tyr | 1 | 140,439 |
| | | 11107481 | C | T | c.907C>T | p.Arg303Trp | 2 | 70,220 |
| | | 11107486 | C | G | c.912C>G | p.Asp304Glu | 4 | 35,110 |
| | | 11107512 | G | A | c.938G>A | p.Cys313Tyr | 2 | 70,220 |
| | | 11110660 | G | A | c.949G>A | p.Glu317Lys | 35 | 4,013 |
| | | 11110678 | G | A | c.967G>A | p.Gly323Ser | 1 | 140,439 |
| | | 11110714 | G | A | c.1003G>A | p.Gly335Ser | 3 | 46,813 |
| | | 11110738 | G | A | c.1027G>A | p.Gly343Ser | 8 | 17,555 |
| | | 11110759 | C | T | c.1048C>T | p.Arg350Ter | 4 | 35,110 |
| | | 11110760 | G | C | c.1049G>C | p.Arg350Pro | 4 | 35,110 |
| | | 11111571 | G | A | c.1118G>A | p.Gly373Asp | 1 | 140,439 |
| | | 11111619 | C | T | c.1166C>T | p.Thr389Met | 8 | 17,555 |
| | | 11113286 | G | A | c.1195G>A | p.Ala399Thr | 1 | 140,439 |
| | | 11113287 | C | A | c.1196C>A | p.Ala399Asp | 1 | 140,439 |
| | | 11113292 | CTCTTC | CTCT | c.1205_1206del | p.Phe403HisfsTer37 | 1 | 140,439 |
| | | 11113307 | C | T | c.1216C>T | p.Arg406Trp | 5 | 28,088 |
| | | 11113308 | G | A | c.1217G>A | p.Arg406Gln | 4 | 35,110 |
| | | 11113313 | G | A | c.1222G>A | p.Glu408Lys | 1 | 140,439 |

| | | | | | | | | |
|---|---|---|---|---|---|---|---|---|
| | | 11113322 | A | G | c.1231A>G | p.Lys411Glu | 1 | 140,439 |
| | | 11113329 | C | T | c.1238C>T | p.Thr413Met | 14 | 10,031 |
| | | 11113337 | C | T | c.1246C>T | p.Arg416Trp | 2 | 70,220 |
| | | 11113419 | G | C | c.1328G>C | p.Trp443Ser | 1 | 140,439 |
| | | 11113426 | C | G | c.1335C>G | p.Asp445Glu | 5 | 28,088 |
| | | 11113554 | CA | C | c.1379del | p.His460ProfsTer47 | 1 | 140,439 |
| | | 11113590 | G | T | c.1414G>T | p.Asp472Tyr | 6 | 23,407 |
| | | 11113608 | G | A | c.1432G>A | p.Gly478Arg | 2 | 70,220 |
| | | 11113612 | T | C | c.1436T>C | p.Leu479Pro | 2 | 70,220 |
| | | 11113620 | G | A | c.1444G>A | p.Asp482Asn | 29 | 4,843 |
| | | 11113650 | G | A | c.1474G>A | p.Asp492Asn | 1 | 140,439 |
| | | 11113678 | C | T | c.1502C>T | p.Ala501Val | 5 | 28,088 |
| | | 11113705 | C | T | c.1529C>T | p.Thr510Met | 3 | 46,813 |
| | | 11113743 | G | A | c.1567G>A | p.Val523Met | 1 | 140,439 |
| | | 11116095 | T | G | c.1588T>G | p.Phe530Val | 10 | 14,044 |
| | | 11116125 | G | A | c.1618G>A | p.Ala540Thr | 2 | 70,220 |
| | | 11116141 | G | A | c.1634G>A | p.Gly545Glu | 1 | 140,439 |
| | | 11116198 | A | G | c.1691A>G | p.Asn564Ser | 2 | 70,220 |
| | | 11116873 | C | T | c.1720C>T | p.Arg574Cys | 2 | 70,220 |
| | | 11116898 | T | C | c.1745T>C | p.Leu582Pro | 1 | 140,439 |
| | | 11116918 | G | A | c.1765G>A | p.Asp589Asn | 1 | 140,439 |

| | | | | | | | | |
|---|---|---|---|---|---|---|---|---|
| | | 11116928 | G | A | c.1775G>A | p.Gly592Glu | 1 | 140,439 |
| | | 11116936 | C | T | c.1783C>T | p.Arg595Trp | 6 | 23,407 |
| | | 11116937 | G | A | c.1784G>A | p.Arg595Gln | 2 | 70,220 |
| | | 11116976 | C | G | c.1823C>G | p.Pro608Arg | 1 | 140,439 |
| | | 11120091 | G | A | c.1846-1G>A | . | 1 | 140,439 |
| | | 11120106 | G | T | c.1860G>T | p.Trp620Cys | 1 | 140,439 |
| | | 11120110 | GAT | G | c.1867_1868del | p.Ile623HisfsTer21 | 1 | 140,439 |
| | | 11120143 | C | T | c.1897C>T | p.Arg633Cys | 9 | 15,604 |
| | | 11120144 | G | A | c.1898G>A | p.Arg633His | 1 | 140,439 |
| | | 11120152 | G | A | c.1906G>A | p.Gly636Ser | 3 | 46,813 |
| | | 11120212 | C | A | c.1966C>A | p.His656Asn | 8 | 17,555 |
| | | 11120370 | G | A | c.1988G>A | p.Gly663Glu | 1 | 140,439 |
| | | 11120408 | G | A | c.2026G>A | p.Gly676Ser | 5 | 28,088 |
| | | 11120436 | C | T | c.2054C>T | p.Pro685Leu | 12 | 11,703 |
| | | 11120441 | A | T | c.2059A>T | p.Ile687Phe | 5 | 28,088 |
| | | 11120442 | T | TC | c.2061dup | p.Asn688GlnfsTer29 | 1 | 140,439 |
| | | 11123200 | G | T | c.2167G>T | p.Glu723Ter | 1 | 140,439 |
| | | 11128027 | C | CA | c.2332dup | p.Arg778LysfsTer4 | 1 | 140,439 |

**Supplemental Table 3. List of variants of unknown significance (VUS) identified in White British participants of the UK Biobank.** Genetic coordinates are mapped to GRCh38. Count refers to the number of participants having the VUS.

| Gene | Chromosome number | Position | Reference allele | Alternate allele | HGVSc | HGVSp | Count |
|---|---|---|---|---|---|---|---|
| *APOB* | 2 | 21001939 | ACTG | A | ENST00000233242:c.13480_13482delCAG | ENSP00000233242.1:p.Gln4494del | 132 |
| | | 21006196 | C | T | ENST00000233242:c.10672C>T | ENSP00000233242.1:p.Arg3558Cys | 299 |
| | | 21006239 | C | G | ENST00000233242:c.10629C>G | ENSP00000233242.1:p.Asn3543Lys | 3 |
| | | 21006349 | C | T | ENST00000233242:c.10519C>T | ENSP00000233242.1:p.Arg3507Trp | 1 |
| | | 21015387 | G | C | ENST00000233242:c.3491G>C | ENSP00000233242.1:p.Arg1164Thr | 1 |
| *PCSK9* | 1 | 55044021 | A | G | ENST00000302118:c.386A>G | ENSP00000303208.5:p.Asp129Gly | 2 |
| | | 55052698 | G | A | ENST00000302118:c.706G>A | ENSP00000303208.5:p.Gly236Ser | 4 |
| | | 55058543 | C | G | ENST00000302118:c.1399C>G | ENSP00000303208.5:p.Pro467Ala | 3 |
| *LDLR* | 19 | 11100261 | G | C | ENST00000558518.6:c.106G>C | ENSP00000454071.1:p.Asp36His | 1 |
| | | 11100322 | C | T | ENST00000558518.6:c.167C>T | ENSP00000454071.1:p.Ser56Phe | 1 |
| | | 11100328 | A | T | ENST00000558518.6:c.173A>T | ENSP00000454071.1:p.Glu58Val | 2 |

| | | | | | | | |
|---|---|---|---|---|---|---|---|
| | | 11100340 | C | T | ENST00000558518.6:c.185C>T | ENSP00000454071.1:p.Thr62Met | 10 |
| | | 11102720 | A | T | ENST00000558518.6:c.247A>T | ENSP00000454071.1:p.Ile83Phe | 1 |
| | | 11105262 | G | C | ENST00000558518.6:c.356G>C | ENSP00000454071.1:p.Gly119Ala | 1 |
| | | 11105337 | C | T | ENST00000558518.6:c.431C>T | ENSP00000454071.1:p.Pro144Leu | 1 |
| | | 11105379 | C | T | ENST00000558518.6:c.473C>T | ENSP00000454071.1:p.Ser158Phe | 1 |
| | | 11105414 | G | A | ENST00000558518.6:c.508G>A | ENSP00000454071.1:p.Asp170Asn | 22 |
| | | 11105415 | AC | GC | ENST00000558518.6:c.509delinsG | ENSP00000454071.1:p.Asp170Gly | 1 |
| | | 11106580 | G | A | ENST00000558518.6:c.710G>A | ENSP00000454071.1:p.Arg237His | 10 |
| | | 11106593 | C | A | ENST00000558518.6:c.723C>A | ENSP00000454071.1:p.Phe241Leu | 3 |
| | | 11106601 | C | G | ENST00000558518.6:c.731C>G | ENSP00000454071.1:p.Ser244Cys | 1 |
| | | 11106639 | C | T | ENST00000558518.6:c.769C>T | ENSP00000454071.1:p.Arg257Trp | 2 |
| | | 11107472 | A | G | ENST00000558518.6:c.898A>G | ENSP00000454071.1:p.Arg300Gly | 1 |
| | | 11111538 | A | C | ENST00000558518.6:c.1085A>C | ENSP00000454071.1:p.Asp362Ala | 60 |
| | | 11111558 | G | A | ENST00000558518.6:c.1105G>A | ENSP00000454071.1:p.Val369Met | 3 |

| | | | | | | | |
|---|---|---|---|---|---|---|---|
| | | 11111609 | G | T | ENST00000558518.6:c.1156G>T | ENSP00000454071.1:p.Asp386Tyr | 5 |
| | | 11113278 | G | T | ENST00000558518.6:c.1187G>T | ENSP00000454071.1:p.Gly396Val | 1 |
| | | 11113287 | C | T | ENST00000558518.6:c.1196C>T | ENSP00000454071.1:p.Ala399Val | 1 |
| | | 11113292 | CTCTTC | CTCTTG | ENST00000558518.6:c.1206delinsG | ENSP00000454071.1:p.Phe402Leu | 1 |
| | | 11113362 | C | T | ENST00000558518.6:c.1271C>T | ENSP00000454071.1:p.Pro424Leu | 3 |
| | | 11113374 | A | C | ENST00000558518.6:c.1283A>C | ENSP00000454071.1:p.Asn428Thr | 1 |
| | | 11113409 | A | G | ENST00000558518.6:c.1318A>G | ENSP00000454071.1:p.Arg440Gly | 4 |
| | | 11113561 | TCTCTTCCTA | TCTCTTACTA | ENST00000558518.6:c.1391delinsA | ENSP00000454071.1:p.Ser464Tyr | 2 |
| | | 11113625 | G | T | ENST00000558518.6:c.1449G>T | ENSP00000454071.1:p.Trp483Cys | 1 |
| | | 11113751 | T | G | ENST00000558518.6:c.1575T>G | ENSP00000454071.1:p.Asp525Glu | 6 |
| | | 11113762 | G | T | ENST00000558518.6:c.1586G>T | ENSP00000454071.1:p.Gly529Val | 1 |
| | | 11116101 | T | C | ENST00000558518.6:c.1594T>C | ENSP00000454071.1:p.Tyr532His | 1 |
| | | 11116132 | T | A | ENST00000558518.6:c.1625T>A | ENSP00000454071.1:p.Ile542Asn | 1 |
| | | 11116205 | C | G | ENST00000558518.6:c.1698C>G | ENSP00000454071.1:p.Ile566Met | 1 |

| | | | | | | | |
|---|---|---|---|---|---|---|---|
| | | 11116885 | G | A | ENST00000558518.6:c.1732G>A | ENSP00000454071.1:p.Val578Ile | 2 |
| | | 11116914 | C | G | ENST00000558518.6:c.1761C>G | ENSP00000454071.1:p.Ser587Arg | 4 |
| | | 11116949 | T | C | ENST00000558518.6:c.1796T>C | ENSP00000454071.1:p.Leu599Ser | 4 |
| | | 11116970 | C | A | ENST00000558518.6:c.1817C>A | ENSP00000454071.1:p.Ala606Asp | 14 |
| | | 11120454 | C | T | ENST00000558518.6:c.2072C>T | ENSP00000454071.1:p.Ser691Leu | 4 |
| | | 11120484 | G | T | ENST00000558518.6:c.2102G>T | ENSP00000454071.1:p.Gly701Val | 1 |
| | | 11120507 | A | G | ENST00000558518.6:c.2125A>G | ENSP00000454071.1:p.Arg709Gly | 1 |
| | | 11123315 | C | T | ENST00000558518.6:c.2282C>T | ENSP00000454071.1:p.Thr761Met | 11 |
| | | 11128062 | C | A | ENST00000558518.6:c.2366C>A | ENSP00000454071.1:p.Ala789Asp | 1 |
| | | 11129553 | G | C | ENST00000558518.6:c.2430G>C | ENSP00000454071.1:p.Trp810Cys | 1 |
| | | 11129573 | A | T | ENST00000558518.6:c.2450A>T | ENSP00000454071.1:p.Asn817Ile | 1 |
| | | 11129582 | G | A | ENST00000558518.6:c.2459G>A | ENSP00000454071.1:p.Ser820Asn | 1 |
| | | 11129633 | A | G | ENST00000558518.6:c.2510A>G | ENSP00000454071.1:p.His837Arg | 18 |
| | | 11129653 | G | A | ENST00000558518.6:c.2530G>A | ENSP00000454071.1:p.Gly844Ser | 1 |

| | | | | | | | |
|---|---|---|---|---|---|---|---|
| | | 11131299 | G | C | ENST00000558518.6:c.2566G>C | ENSP00000454071.1:p.Glu856Gln | 1 |

**Supplemental Table 4. Participant characteristic comparison between the UK Biobank participants of our study cohort and the NHS Health Check 2017-2018.**[10]

| | NHS Health Check (2017-2018) | UK Biobank study cohort |
|---|---|---|
| Count of participants | 1,108,841 | 140,439 |
| Sex (male) | 509,752 (46.0%) | 63878 (45.5%) |
| Age | | |
| 39 | 0 (0%) | 2 (0.001%) |
| 40 to 44 | 240,438 (21.7%) | 13,338 (9.5%) |
| 45 to 49 | 205,722 (18.6%) | 17,577 (12.5%) |
| 50 to 54 | 209,088 (18.9%) | 21,066 (15.0%) |
| 55 to 59 | 180,624 (16.3%) | 25,385 (18.1%) |
| 60 to 64 | 147,444 (13.3%) | 35,473 (25.3%) |
| 65 to 69 | 125,525 (11.3%) | 26,938 (19.2%) |
| 70 to 74 | 0 (0%) | 660 (0.5%) |
| Self-reported ethnicity | | |
| Any other ethnic group | 17,531 (1.6%) | 0 (0%) |
| Asian or Asian British | 98,692 (8.9%) | 0 (0%) |
| Black or African or Caribbean or Black British | 45,674 (4.1%) | 0 (0%) |
| Mixed or multiple ethnic groups | 13,498 (1.2%) | 0 (0%) |
| White | 864,173 (77.9%) | 140,439 (100%) |
| Ethnicity not stated or recorded | 69,273 (6.2%) | 0 (0%) |

**Supplemental Table 5. Study participants characteristics categorised by FH-causing gene.** Missing (%) refers to the proportion of missing data in each field. IQR = interquartile range; BMI = body mass index; CHD = coronary heart disease; LDL-C = low-density lipoprotein cholesterol; HDL-C = high-density lipoprotein cholesterol; CVD = cardiovascular disease (defined as CHD, ischaemic and haemorrhagic stroke, heart failure, and atrial fibrillation).

| | *LDLR* | *APOB* | *APOE* | P-value of group differences | Missing (%) |
|---|---|---|---|---|---|
| n | 374 | 101 | 13 | | |
| Age (median [IQR]) | 58.00 [51.00, 63.00] | 57.00 [51.00, 62.00] | 63.00 [53.00, 66.00] | 0.378 | 0.0 |
| Sex (male) (%) | 156 (41.7) | 45 (44.6) | 6 (46.2) | 0.844 | 0.0 |
| Townsend deprivation index (median [IQR]) | -2.22 [-3.64, 0.16] | -2.20 [-3.98, 0.13] | -1.37 [-3.47, -0.12] | 0.848 | 0.4 |
| Smoking status (%) | | | | 0.825 | 0.0 |
| Non-smoker | 217 (58.0) | 59 (58.4) | 5 (38.5) | | |
| Former smoker | 131 (35.0) | 35 (34.7) | 7 (53.8) | | |
| Light smoker (<10 cigarettes/day) | 5 (1.3) | 2 (2.0) | 0 (0.0) | | |
| Moderate smoker (10-19 cigarettes/day) | 10 (2.7) | 2 (2.0) | 1 (7.7) | | |
| Heavy Smoker (>20 cigarettes/day) | 11 (2.9) | 3 (3.0) | 0 (0.0) | | |
| BMI, kg/m2 (median [IQR]) | 26.92 [23.84, 29.61] | 27.78 [24.02, 30.06] | 25.73 [24.81, 27.26] | 0.245 | 0.2 |
| Family history of CHD (%) | 237 (63.4) | 61 (60.4) | 8 (61.5) | 0.857 | 0.0 |
| Statin use (%) | 128 (34.2) | 33 (32.7) | 4 (30.8) | 0.932 | 0.0 |
| **Biomarkers** | | | | | |
| LDL-C (unadjusted), mmol/L (median [IQR]) | 3.74 [3.05, 4.71] | 4.35 [3.81, 5.32] | 3.55 [3.01, 4.25] | <0.001 | 0.0 |
| LDL-C (adjusted for statin users), mmol/L (median [IQR]) | 4.28 [3.56, 5.23] | 5.01 [4.28, 5.76] | 3.68 [3.55, 4.98] | <0.001 | 0.0 |
| HDL-C, mmol/L (median [IQR]) | 1.38 [1.18, 1.64] | 1.34 [1.15, 1.58] | 1.73 [1.50, 1.86] | 0.037 | 14.5 |
| Total cholesterol, mmol/L (median [IQR]) | 5.93 [5.06, 7.02] | 6.56 [5.70, 8.03] | 5.58 [5.21, 6.58] | 0.001 | 6.1 |
| Triglycerides, mmol/L (median [IQR]) | 1.28 [0.92, 1.91] | 1.26 [1.00, 1.93] | 0.76 [0.69, 1.13] | 0.019 | 6.1 |

| | | | | | |
|---|---|---|---|---|---|
| Lipoprotein(a), nmol/L (median [IQR]) | 29.00 [11.52, 60.52] | 26.35 [8.52, 50.35] | 8.50 [4.77, 57.71] | 0.245 | 26.8 |
| Apolipoprotein A, g/L (median [IQR]) | 1.46 [1.33, 1.64] | 1.44 [1.26, 1.60] | 1.67 [1.56, 1.75] | 0.029 | 15.0 |
| Apolipoprotein B, g/L (median [IQR]) | 1.11 [0.96, 1.34] | 1.31 [1.12, 1.46] | 0.99 [0.81, 1.16] | <0.001 | 7.2 |
| C-reactive protein, mg/L (median [IQR]) | 1.19 [0.58, 2.20] | 1.25 [0.69, 2.64] | 1.45 [0.76, 1.93] | 0.402 | 6.4 |
| **Disease prevalence & incidence** | | | | | |
| CHD prevalence (%) | 30 (8.0) | 10 (9.9) | 0 (0.0) | 0.457 | 0.0 |
| CVD prevalence (%) | 34 (9.1) | 11 (10.9) | 0 (0.0) | 0.435 | 0.0 |
| Type 2 diabetes prevalence (%) | 10 (2.7) | 1 (1.0) | 0 (0.0) | 0.514 | 0.0 |

**Supplemental Table 6. The counts obtained from the two-stage screen in our study cohort of 140,439 individuals for various LDL-C cut-off values.** OAPR = odds of being affected given a positive test result; VUS = variant of unknown significance.

| Cut-off | Detection rate (sensitivity) | False positive rate (1-specificity) | Positive predictive value (PPV) | Negative predictive value (NPV) | OAPR | Cases missed | True positive cases | False positive cases | Number sent for sequencing | Number of VUS above threshold |
|---|---|---|---|---|---|---|---|---|---|---|
| 3 | 90.6 (87.7-92.9) | 80.5 (80.3-80.7) | 0.4 (0.4-0.4) | 99.8 (99.8-99.9) | 1:255 | 46 | 442 | 112713 | 113155 | 586 |
| 3.5 | 79.7 (75.9-83) | 58.5 (58.3-58.8) | 0.5 (0.4-0.5) | 99.8 (99.8-99.9) | 1:211 | 99 | 389 | 81940 | 82329 | 465 |
| 4 | 64.8 (60.4-68.9) | 34.8 (34.6-35) | 0.6 (0.6-0.7) | 99.8 (99.8-99.8) | 1:154 | 172 | 316 | 48702 | 49018 | 306 |
| 4.1 | 61.5 (57.1-65.7) | 30.7 (30.4-30.9) | 0.7 (0.6-0.8) | 99.8 (99.8-99.8) | 1:143 | 188 | 300 | 42913 | 43213 | 275 |
| 4.2 | 59.2 (54.8-63.5) | 26.7 (26.5-27) | 0.8 (0.7-0.9) | 99.8 (99.8-99.8) | 1:129 | 199 | 289 | 37396 | 37685 | 248 |
| 4.3 | 54.1 (49.7-58.5) | 23.2 (22.9-23.4) | 0.8 (0.7-0.9) | 99.8 (99.8-99.8) | 1:123 | 224 | 264 | 32406 | 32670 | 223 |
| 4.4 | 50.8 (46.4-55.2) | 19.9 (19.7-20.1) | 0.9 (0.8-1) | 99.8 (99.8-99.8) | 1:112 | 240 | 248 | 27856 | 28104 | 195 |
| 4.5 | 47.7 (43.3-52.2) | 17.1 (16.9-17.3) | 1 (0.9-1.1) | 99.8 (99.8-99.8) | 1:102 | 255 | 233 | 23871 | 24104 | 174 |
| 4.6 | 45.5 (41.1-49.9) | 14.5 (14.3-14.7) | 1.1 (0.9-1.2) | 99.8 (99.7-99.8) | 1:92 | 266 | 222 | 20322 | 20544 | 151 |
| 4.7 | 43.2 (38.9-47.7) | 12.2 (12.1-12.4) | 1.2 (1.1-1.4) | 99.8 (99.7-99.8) | 1:81 | 277 | 211 | 17127 | 17338 | 129 |
| 4.8 | 39.8 (35.5-44.2) | 10.3 (10.1-10.5) | 1.3 (1.2-1.5) | 99.8 (99.7-99.8) | 1:74 | 294 | 194 | 14409 | 14603 | 111 |
| 4.9 | 37.7 (33.5-42.1) | 8.6 (8.5-8.7) | 1.5 (1.3-1.7) | 99.8 (99.7-99.8) | 1:65 | 304 | 184 | 12038 | 12222 | 99 |
| 5 | 34.8 (30.7-39.2) | 7.1 (7-7.3) | 1.7 (1.4-1.9) | 99.8 (99.7-99.8) | 1:59 | 318 | 170 | 9994 | 10164 | 83 |
| 6.8 | 3.9 (2.5-6) | 0.2 (0.2-0.2) | 6 (3.9-9.2) | 99.7 (99.6-99.7) | 1:16 | 469 | 19 | 297 | 316 | 2 |
| 8.5 | 0.4 (0.1-1.5) | 0 (0-0) | 8.7 (2.4-26.8) | 99.7 (99.6-99.7) | 1:10 | 486 | 2 | 21 | 23 | 0 |

**Supplemental Figure 1. The detection rate (DR) and false positive rate (FPR) depicted as a receiver operating characteristic (ROC) curve for various LDL-C cut-points.** The AUC (95% confidence intervals) of the ROC curve is specified on the figure.

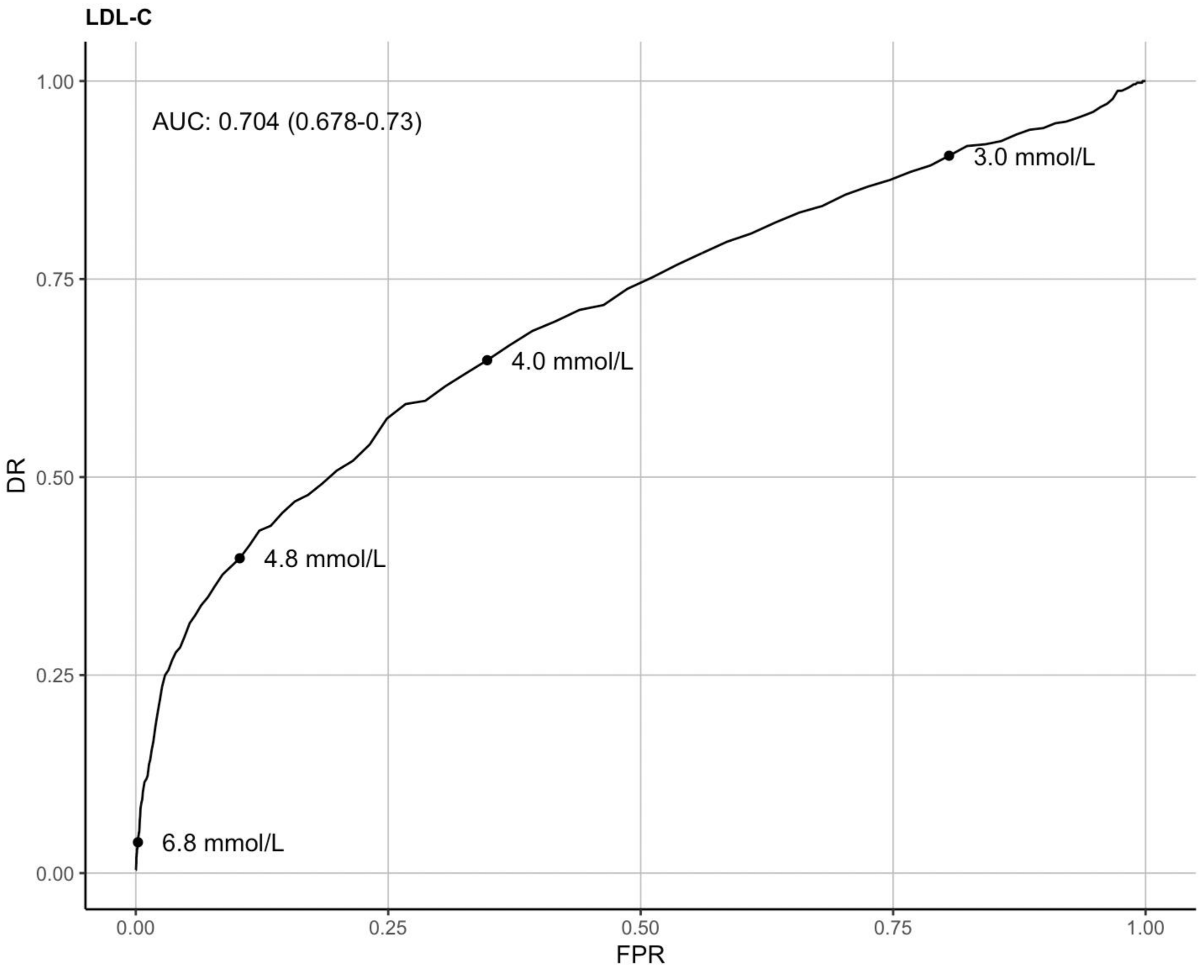

Supplement: online supplemental file 1 [file bmjph-1-1-s001.pdf]
